# Biodegradable Nanoparticles Encapsulating Murine Double Minute 2 siRNA to Treat Peritoneal Dissemination of Colon Cancer

**DOI:** 10.3390/ijms26188883

**Published:** 2025-09-12

**Authors:** Tomoaki Kurosaki, Akari Okada, Yuuki Takashima, Hitoshi Sasaki, Yukinobu Kodama

**Affiliations:** 1 Institute of Tropical Medicine, Nagasaki University, 1-12-4 Sakamoto, Nagasaki 852-8523, Japan; jj20250042@ms.nagasaki-u.ac.jp (T.K.); sasaki@nagasaki-u.ac.jp (H.S.); 2 Faculty of Pharmacy, Juntendo University, 6-8-1, Hinode, Urayasu 279-0013, Japan; y.takashima.ex@juntendo.ac.jp; 3 Department of Hospital Pharmacy, Nagasaki University Hospital, 1-7-1 Sakamoto, Nagasaki 852-8501, Japan; chiyaku.labo@gmail.com

**Keywords:** siRNA delivery system, biodegradable nanoparticles, colorectal cancer peritoneal dissemination, murine double minute 2

## Abstract

The study aim was to apply murine double minute 2 (MDM2)-siRNA to a biodegradable siRNA delivery vector, ternary complex, for treating colorectal cancer peritoneal dissemination. The ternary complex containing MDM2-siRNA (MDM2-siRNA complex) was constructed by mixing MDM2-siRNA, dendrigraft poly-L-lysine, and γ-polyglutamic acid. Cellular uptake of the ternary complex and suppressive effect on MDM2-mRNA were determined in a mouse colorectal cancer cell line. Tumor-growth inhibition by the MDM2-siRNA complex was evaluated in peritoneal dissemination model mice. The MDM2-siRNA complex, with an approximately 177 nm particle size and −35 mV ζ-potential, prevented degradation of the inner siRNA by RNase. In the in vitro study, the ternary complex was highly taken up by the cells, and 2 μg/mL of the MDM2-siRNA complex significantly decreased MDM2-mRNA to about 30% of control cells. Intraperitoneal administration in colorectal cancer peritoneal dissemination model mice showed little effect of the ternary complex containing scramble-siRNA on cancer growth in the peritoneal cavity. Conversely, the MDM2-siRNA complex significantly reduced peritoneal dissemination to less than 1/1000th of control mice and successfully prolonged survival time. In this study, we found that the biodegradable MDM2-siRNA complex had a suppressive effect on MDM2-mRNA in cancer cells and tumor growth of peritoneal dissemination.

## 1. Introduction

Colorectal cancer is the second most common cancer in women and third most common in men worldwide [1], and the peritoneum is the second most common site of recurrence after the liver [2]. As peritoneal dissemination progresses, it creates an array of symptoms ranging from the exhausted condition resulting from a lack of food and water and intractable ascites to pain and bowel obstruction, resulting ultimately in the affected patient’s death [3]. The clinical diagnosis of peritoneal dissemination is often complicated, and detection is delayed because of its small, flat peritoneal lesions [4]. Currently, various treatments, such as systemic chemotherapy, radiotherapy, and surgical resection, are administered. Peritoneal dissemination, however, is resistant to systemic chemotherapy, with a 5-year survival of only 11% [3,5]. In recent clinical studies, cytoreductive surgery combined with hyperthermic intraperitoneal chemotherapy, which involves direct application of an anti-cancer drug inside the abdominal cavity after surgical removal, prolongs the survival time of colorectal cancer peritoneal dissemination [2]. On the other hand, these therapeutic effects are limited and cause severe adverse reactions. Therefore, new treatment approaches for peritoneal dissemination are needed.

It is well known that tumor protein 53 (p53) is a tumor suppressor in various diseases, including colorectal cancer [6], and suppresses tumor formation and protects DNA against damage by inducing cell-cycle arrest, DNA repair, and apoptosis [7]. The p53 pathway, however, is often mutated in cancer, especially in colorectal cancer [8,9]. Murine double minute 2 (MDM2) is a known physiological regulator of p53 and controls p53 protein levels [7]. In colorectal cancer, MDM2 is overexpressed and degrades p53 by catalyzing ubiquitination [10]. Reportedly, cancer growth was inhibited, including by apoptosis, by MDM2 inhibitor and siRNA through reactivation of the p53 pathway [11,12].

In a previous study, we successfully developed a new siRNA vector, a ternary complex constructed with siRNA, dendrigraft poly-L-lysine (DGL), and γ-polyglutamic acid (γ-PGA) [13]. The complex included firefly luciferase siRNA and strongly suppressed the expression of luciferase without cytotoxicity in a mouse colorectal cancer cell line constitutively expressing luciferase. Peritoneal injection of this ternary complex that included MDM2-siRNA would be useful for treatment of colorectal cancer peritoneal dissemination. Therefore, the present study aimed to prepare a ternary complex containing MDM2-siRNA (MDM2-siRNA complex) and investigate its anti-tumor effect in peritoneal dissemination model mice.

## 2. Results

### 2.1. Physicochemical Characteristics of siRNA Complexes

The DGL solution was mixed with MDM2-siRNA or scramble-siRNA solution to prepare each binary complex. The γ-PGA solution was added to those binary complexes by pipetting to construct the MDM2-siRNA/DGL/γ-PGA complex (MDM2-siRNA complex) or scramble-siRNA/DGL/γ-PGA complex (scramble-siRNA complex). Particle sizes (Z-average) and ζ-potentials of those ternary complexes were approximately 170–180 nm and −35 mV, respectively (Table 1).

### 2.2. Stability of MDM2-siRNA Complex Against RNase A

**Figure 1:**
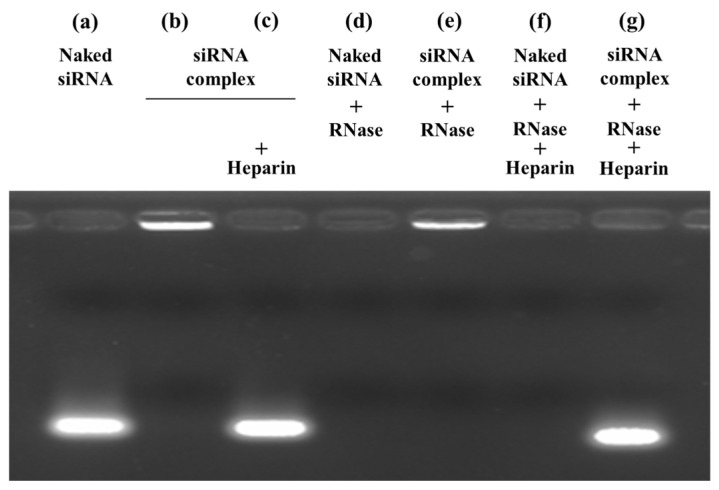
Stability of the naked murine double minute 2 (MDM2)-siRNA and MDM2-siRNA complex against RNase A. The naked MDM2-siRNA or MDM2-siRNA complex were incubated with 5% glucose (a: naked MDM2-siRNA, b: MDM2-siRNA complex, c: MDM2-siRNA complex + heparin) or RNase A (d: naked MDM2-siRNA + RNase A, e: MDM2-siRNA complex + RNase A, f: naked MDM2-siRNA + RNase A + heparin, g: MDM2-siRNA complex + RNase A + heparin). Each sample was loaded onto agarose gel, and electrophoresis was carried out.

Figure 1 shows the results of agarose gel electrophoresis for the naked MDM2-siRNA and MDM2-siRNA complex. The naked MDM2-siRNA and MDM2-siRNA complex were incubated with RNase A for 1 h. Heparin sulfate was added immediately prior to electrophoresis to release MDM2-siRNA from the complex. Naked MDM2-siRNA was detected as a clear band on agarose gel (lane a), but the band disappeared after addition of RNase A with or without heparin sulfate (lanes d and f). In the MDM2-siRNA complex lanes, the band of siRNA was not detected (lane b). After dissociation of the MDM2-siRNA complex by addition of heparin sulfate, the band of siRNA was detected (lane c). After incubation of the MDM2-siRNA complex with RNase A, the band of siRNA was not detected without heparin sulfate (lane e), but siRNA was detected as a band after dissociation of the MDM2-siRNA complex by addition of heparin sulfate (lane g).

### 2.3. Cellular Uptake of siRNA Complex

**Figure 2:**
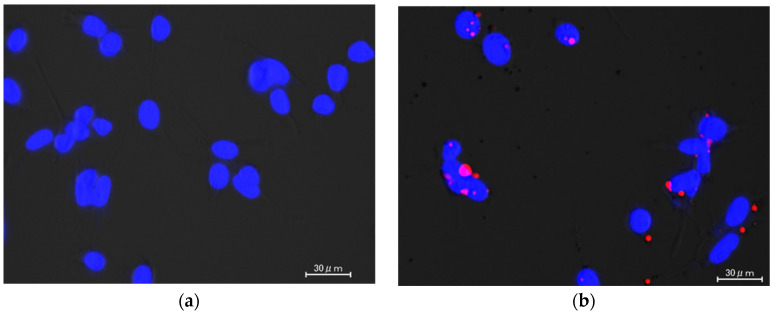
Cellular uptake of the Alexa Fluor 555-siRNA complex. Colon26/Luc cells treated with naked Alexa Fluor 555-siRNA (**a**) and the Alexa Fluor 555-siRNA complex (**b**). The nucleus stained with Hoechst 33,342 (blue). The fluorescence of the Alexa Fluor 555-siRNA observed under a fluorescence microscope is shown in red.

To evaluate the cellular uptake of siRNA, a commercially available fluorescence-labeled negative control siRNA (Alexa Fluor 555-siRNA) was used. The intracellular Alexa Fluor 555-siRNA was observed using a fluorescent microscope. When naked Alexa Fluor 555-siRNA was added, no fluorescence of Alexa Fluor 555-siRNA was observed in the Colon26/Luc cells (Figure 2a). On the other hand, a large amount of red fluorescence of Alexa Fluor 555-siRNA was observed in the cells treated with the Alexa Fluor 555-siRNA complex (Figure 2b).

### 2.4. In Vitro MDM2 Silencing Efficiency of the MDM2-siRNA Complex

**Figure 3:**
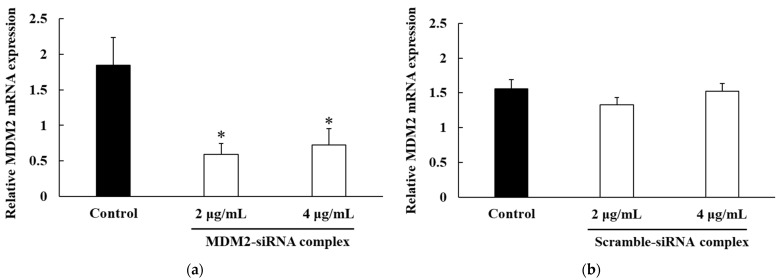
Murine double minute 2 (MDM2)-mRNA silencing effect of the MDM2-siRNA complex. The relative MDM2-mRNA expression level of the Colon26/Luc cells was determined by real-time PCR at 24 h after treatment with MDM2-siRNA (**a**) or scramble-siRNA complex (**b**). Each bar represents the mean + S.D. (*n* = 3). *; *p* < 0.05 vs. control.

The silencing effect of the MDM2-siRNA complex in Colon26/Luc cells was evaluated by performing real-time polymerase chain reaction (PCR) (Figure 3a). The relative MDM2-mRNA expression level was significantly decreased by addition of MDM2-siRNA complex to 32.1–39.3% of the control (*p* < 0.05). At the same time, scramble-siRNA complex showed little effect on the MDM2 mRNA level in the Colon26/Luc cells as shown in Figure 3b.

### 2.5. In Vivo Tumor-Growth Inhibition of the MDM2-siRNA Complex

**Figure 4:**
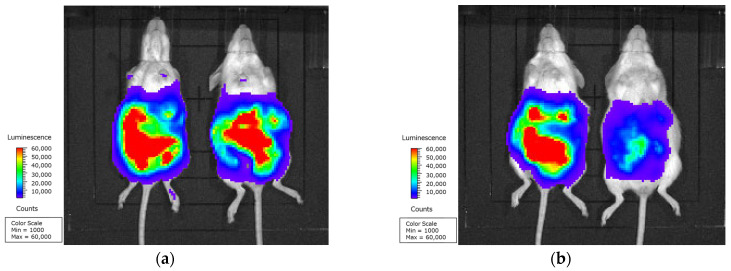

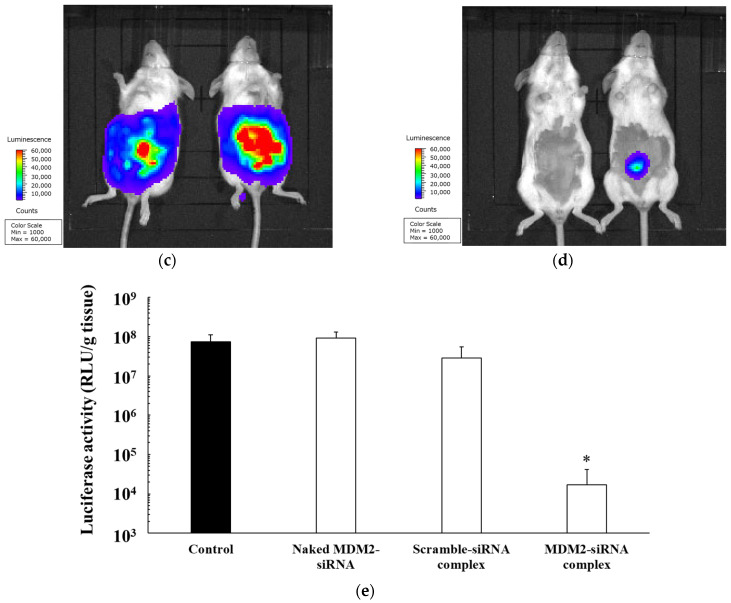
Tumor-growth inhibition in mice treated with the murine double minute 2 (MDM2)-siRNA complex. The Colon26/Luc cells (3.0 × 10^5^ cells/mouse) were injected intraperitoneally into mice. One day after the inoculation, the mice were intraperitoneally injected with the naked MDM2-siRNA, MDM2-siRNA complex, and scramble-siRNA complex. Each formulation includes 20 μg siRNA at a volume of 200 μL per mouse. A 5% glucose solution was injected into mice as a control. At post-administration day 16, two mice were randomly selected from each group and intraperitoneally administrated with 12 mg of luciferin in 300 μL of PBS. The luminescence from the mice injected with the 5% glucose solution (**a**), naked MDM2-siRNA (**b**), scramble-siRNA complex (**c**), and MDM2-siRNA complex (**d**) was monitored on an in vivo imaging system (IVIS) for 30 min. The luciferase activity was determined from homogenates of intestines and clumps of Colon26/Luc cells in the peritoneal cavity (**e**). Each bar represents the mean + S.D. (*n* = 4–5). *; *p* < 0.05 vs. control.

Tumor-growth inhibition of the MDM2-siRNA complex was determined in the peritoneal dissemination model mice. The 5% glucose solution (a), naked MDM2-siRNA (b), scramble-siRNA complex (c), and MDM2-siRNA complex (d) were intraperitoneally administered to the mice. At post-administration day 16, two mice were randomly selected from each group and the growth and metastasis of Colon26/Luc cells in the mouse whole body were observed using an in vivo imaging system. Observation showed that the luminescence from Colon26/Luc cells was observed only around the peritoneal cavity and decreased markedly only in the mice treated with the MDM2-siRNA complex (Figure 4a–d).

As Colon26/Luc cells were increased only in the peritoneal cavity, the intestine and clumps of Colon26/Luc cells were dissected, and the growth of Colon26/Luc cells was quantitatively determined by luciferase assay (Figure 4e). The luciferase activity in the control was 7.44 × 10^7^ RLU/g tissue, and the activity in the mice injected with the MDM2-siRNA complex was significantly decreased (*p* < 0.05). At the same time, there was no suppressive effect in the mice administered the naked MDM2-siRNA and scramble-siRNA complex.

### 2.6. Effect of the MDM2-siRNA Complex on Survival Time in Tumor-Bearing Mice

**Figure 5:**
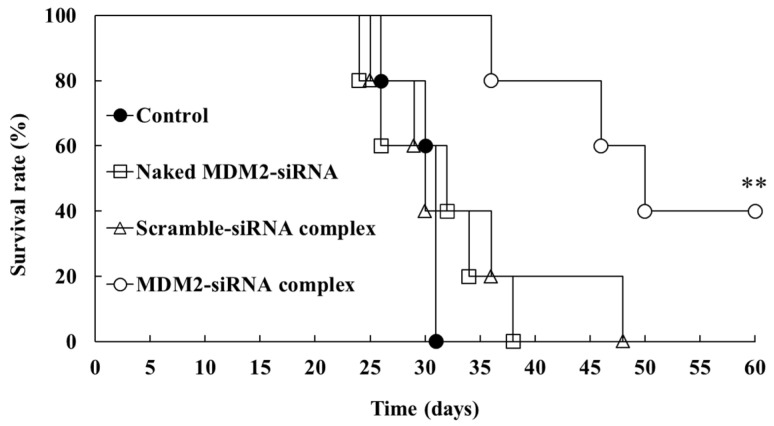
Survival rate of the mice after treatment with the murine double minute 2 (MDM2)-siRNA complex. The Colon26/Luc cells (3.0 × 10^5^ cells/mouse) were injected intraperitoneally into mice. One day after the inoculation, the mice were intraperitoneally injected with the naked MDM2-siRNA, MDM2-siRNA complex, and scramble-siRNA complex and monitored for 60 days after Colon26/Luc cells inoculation (*n* = 5). **; *p* < 0.01 vs. control.

The survival time was monitored ≤60 days after inoculation with Colon26/Luc cells (Figure 5). All control mice died within 31 days after cancer-cell inoculation. Intraperitoneal injection of the MDM2-siRNA complex significantly prolonged the survival time of the peritoneal dissemination model mice (*p* < 0.01). However, the survival time of the mice administered the naked MDM2-siRNA and scramble-siRNA complex was not significantly different from that of the control mice.

## 3. Discussion

As is well known, siRNA is a small molecule with double-stranded RNA that triggers post-transcriptional silencing of target gene expression via RNA interference (RNAi) [11]. Several reports have suggested that siRNA targeting MDM2 could downregulate MDM2 and upregulate p53 [14]. On the other hand, siRNA is unstable in vivo and is degraded rapidly by ribonucleases (RNases), such as the RNase A family [15].

We previously developed an anionic ternary complex containing siRNA, DGL, and γ-PGA that self-assembled through electrostatic interactions. Cationic dendrimers, such as DGL, are theoretically single-molecule compounds that are biodegradable despite their macromolecular size [16]. The molecule γ-PGA has no known toxicity or immunogenicity, so it is used in many biomedical applications against infection, inflammation, cancer, and other conditions and diseases [17,18]. Therefore, we applied the ternary complex to MDM2-siRNA and constructed a MDM2-siRNA complex. This complex has nano-sized particles (177.1 nm) with negatively charged surfaces (−34.9 mV) (Table 1).

Reportedly, siRNA can be rapidly degraded in vivo by RNase A (originally known as pancreatic RNase) [19]. In this study, we evaluated the stability of the MDM2-siRNA complex against RNase A. Although naked MDM2-siRNA was degraded in the presence of RNase A, MDM2-siRNA was encapsulated into the complex and not degraded by RNase A (Figure 1). The blood concentration of RNase A reportedly increased in patients with cancer, which suggests that RNase A in ascites are also increased [20]. In this experiment, the high stability of the MDM2-siRNA complex in ascites suggested that the MDM2-siRNA complex would be suitable for intraperitoneal administration.

We also measured the in vitro uptake of the Alexa Fluor 555-siRNA complex in colorectal cancer Colon26/Luc cells (Figure 2). Generally, anionic polymers, such as siRNA and pDNA, have low intracellular uptake efficiency because of their electrostatic repulsion with negatively charged cellular membranes. In our previous study, however, we confirmed that a ternary complex constructed with siRNA, DGL, and γ-PGA showed good cellular uptake and a high silencing effect despite its negative charge [13]. It has also been reported that γ-PGA-coated anionic nanoparticles were taken up by cells through the specific route involving γ-PGA [21]. In this study, the high cellular uptake of the Alexa Fluor 555-siRNA complex was also determined in the Colon26/Luc cells (Figure 2). However, very low cellular uptake of naked siRNA was also observed.

The in vitro MDM2-mRNA suppressing effect of the MDM2-siRNA complex and scramble-siRNA complex in Colon26/Luc cells was determined by real-time PCR (Figure 3). As shown in Figure 3b, the addition of the scramble-siRNA complex to Colon26/Luc cells did not decrease MDM2-mRNA expression. We have confirmed that naked luciferase-siRNA had little effect on luciferase-mRNA expression in Colon26/Luc cells in the preliminary experiment. On the other hand, the MDM2-siRNA complex significantly decreased MDM2-mRNA expression at concentrations of 2 μg/mL and 4 μg/mL (*p* < 0.05). It is well known that p53 induces cell apoptosis, which blocks the cell cycle at the G1/S phase after suppressing MDM2 [22]. We suggest that the MDM2-siRNA complex should be taken up by cancer cells in the peritoneal cavity, suppress MDM2-mRNA expression, and induce cancer-cell apoptosis in vivo.

Therefore, in vivo tumor-growth inhibition by the MDM2-siRNA complex was evaluated in colorectal cancer peritoneal dissemination model mice, which were intraperitoneally injected with Colon26/Luc cells (Figure 4). The intraperitoneal administration of the MDM2-siRNA complex significantly decreased the tumor growth in the mice peritoneum post-administration day 16. Similarly, the survival time of the colorectal cancer peritoneal dissemination model mice was prolonged only by administering the MDM2-siRNA complex (Figure 5). However, assessing the off-target effect can be a problem in studies using siRNA [23]. The naked MDM2-siRNA and scramble-siRNA complex had little effect on the tumor growth and survival time, suggesting that the observed tumor-growth inhibition by the MDM2-siRNA complex was due to silencing of MDM2 mRNA, not to an off-target effect.

In this study, we found that the MDM2-siRNA complex had a suppressive effect on MDM2-mRNA and tumor growth of peritoneal dissemination. Further study would be needed to demonstrate selective uptakes of MDM2-siRNA complex by Colon26/Luc cells in peritoneal cavity and evaluate how to apply the MDM2-siRNA complex in clinical therapy.

## 4. Materials and Methods

### 4.1. Chemicals

MDM2-siRNA (sense: 5′-GCUUCGGAACAAGAGACUC-3′, antisense: 5′-GAGUCUCUUGUUCCGAAGC-3′) and scramble-siRNA (sense: 5′-CUUACGCUGUCAUGAUCGA-3′, antisense: 5′-UCGAUCAUGACAGCGUAAG-3′) were purchased from GeneDesign, Inc. (Osaka, Japan). A commercially available Alexa Fluor 555-siRNA (BLOCK-iT Alexa Fluor Red Fluorescent Control; the siRNA sequence is not publicly accessible) was obtained from Invitrogen (Carlsbad, CA, USA). Fifth-generation DGL compounds (MW: 172,300 Da, 963 lysine groups) was obtained from COLCOM S.A.S. (Montpellier, France). The compound γ-PGA was kindly provided by Yakult Pharmaceutical Industry Co., Ltd. (Tokyo, Japan). Bisbenzimide H 33,342 trihydrochloride (Hoechst 33,342) was obtained from Sigma Aldrich (St. Louis, MO, USA). Fetal bovine serum (FBS) was purchased from Biological Industries Ltd. (Kibbutz Beit Haemek, Israel). RPMI 1640, Opti-MEM, antibiotics (100 U/mL penicillin and 100 μg/mL streptomycin), and other culture reagents were obtained from GIBCO BRL (Grand Island, NY, USA). A solution of the antibiotic, G418, was purchased from Roche Diagnostics (Indianapolis, IN, USA).

### 4.2. Preparation of the Complex

The siRNA, DGL, and γ-PGA were dissolved in a 5% glucose solution at a concentration of 10 mg/mL. A theoretical charge ratio of the phosphate of siRNA, nitrogen of DGL, and carboxylate of γ-PGA was calculated for complex formation. To prepare the binary complex, an appropriate amount of stock DGL solution was mixed with siRNA solution at a charge ratio of 1:6 by pipetting, and placed on ice for 10 min. After 10 min, the binary complex was maintained at room temperature for 20 min. The γ-PGA solution was added to the binary complex by pipetting to give a charge ratio of 1:6:10 (siRNA:DGL:γ-PGA) to construct the MDM2-siRNA/DGL/γ-PGA complex (MDM2-siRNA complex) or scramble-siRNA/DGL/γ-PGA complex (scramble-siRNA complex), both of which were maintained for another 30 min at room temperature.

### 4.3. Cell Culture

The mouse colorectal cancer cell line expressing luciferase regularly (Colon26/Luc cells) was prepared in our laboratory [13]. The cells were incubated in RPMI 1640 supplemented with 10% FBS and antibiotics (culture medium) under a humidified atmosphere of 5% CO_2_ in air at 37 °C.

### 4.4. Animals

All animal care and experimental procedures were performed according to the Guidelines for Animal Experimentation of Nagasaki University, with approval from the Institutional Animal Care and Use Committee of Nagasaki University. Female 5-week-old BALB/c mice were purchased from Japan SLC, Inc. (Shizuoka, Japan). At least five mice are required per group for tumor-growth experiments. We used total 40 mice in this manuscript. After delivery, the mice were acclimatized to their new environment for ≥1 day before the experiments. All animals were housed under a 12 h dark–light cycle with ad libitum food and water.

### 4.5. Physicochemical Characteristics of Complex

A Zetasizer Nano ZS (Malvern Instruments, Ltd., Malvern, UK) was used to measure the particle size and ζ-potential of the MDM2-siRNA complex and scramble-siRNA complex. Particle sizes are shown as the Z-average particle size.

### 4.6. Agarose Gel Electrophoresis

To determine the complex formation and degradation of siRNA by RNase A, the naked MDM2-siRNA and MDM2-siRNA complex were incubated with and without 0.05 mg/mL RNase A for 1 h. To release MDM2-siRNA from the ternary complex, heparin sulfate was added to the solution after the incubation. Immediately after the additions of heparin sulfate, 20 μL aliquots of the solution containing 1 μg of MDM2-siRNA were mixed with 4 μL of loading buffer (30% glycerol and 0.2% bromophenol blue) and then loaded onto 1% agarose gel. Electrophoresis (i-Mupid J; Cosmo Bio, Tokyo, Japan) was performed at 100 W in a running buffer solution (40 mM Tris/HCl, 40 mM acetic acid, and 1 mM ethylenediaminetetraacetic acid [EDTA]) for 30 min, and siRNA retardation was visualized with ethidium bromide staining on a Gel Doc EZ Imager (Bio-Rad, Hercules, CA, USA).

### 4.7. In Vitro Cellular Uptake of Complex

The Colon26/Luc cells were plated on 24-well plates at a density of 1.0 × 10^4^ cells/well and cultivated in 500 µL of culture medium. After 24 h pre-incubation, the cells were treated with naked Alexa Fluor 555-siRNA and Alexa Fluor 555-siRNA complex at a siRNA concentration of 2 μg/mL to visualize the uptake of Alexa Fluor 555-siRNA. After a 2 h incubation, the cells were treated with 2 μg/mL Hoechst 33,342 solution for 15 min at 37 °C in the dark. A BZ-X Analyzer (KEYENCE, Osaka, Japan) was used to observe the fluorescence of the Alexa Fluor 555-siRNA.

### 4.8. In Vitro MDM2 Silencing Efficiency

The Colon26/Luc cells were plated on 24-well plates at a density of 1.0 × 10^4^ cells/well and cultivated in 500 µL of culture medium. After 24 h pre-incubation, the cells were treated with 2 μg/mL or 4 μg/mL MDM2-siRNA complex or scramble-siRNA complex for 2 h. After treatment, the medium was replaced with fresh culture medium, and the cells were then cultured for 22 h at 37 °C. After 22 h, an ISOGEN II (Nippon Gene, Tokyo, Japan) was used to isolate the total RNA. A PrimeScript RT Master Mix (Takara Bio, Shiga, Japan) was used to reverse transcribe the RNA to cDNA. Real-time PCR was performed with TBGREEN Premix Ex Taq II (Takara Bio) by an AriaMx Real-Time PCR System (Agilent Technologies, Santa Clara, CA, USA) using the primers listed in Table 2. The MDM2-mRNA expression data were normalized to the expression of glyceraldehyde-3-phosphate dehydrogenase (GAPDH)-mRNA.

### 4.9. In Vivo Tumor-Growth Inhibition

In vivo tumor-growth inhibition by the MDM2-siRNA complex was evaluated in peritoneal dissemination model mice. The Colon26/Luc cells (3.0 × 10^5^ cells/mouse) were injected intraperitoneally into the mice. The randomization occurred one day after the injection of Colon26/Luc cells. All of the mice were placed in the same cage and selected at random. After the randomization, those mice were intraperitoneally injected with the naked MDM2-siRNA (*n* = 5), MDM2-siRNA complex (*n* = 5), and scramble-siRNA complex (*n* = 5). Each formulation included 20 μg siRNA at a volume of 200 μL per mouse. A 5% glucose solution was injected into the mice as a control (*n* = 5). T.K. and A.O. were aware of the group allocation after those randomization and injection. At post-administration day 16, 12 mg of luciferin in 300 μL of PBS was injected intraperitoneally into the mice to assess the tumor growth in the mouse whole body by the expression of luciferase. The luminescence intensity from the mice was observed for 30 min by an in vivo imaging system (Xenogen IVIS Lumina System; Xenogen Co., Alameda, CA, USA) with the Living Image 4.0 software (Xenogen Co.) used for data acquisition under anesthesia using medetomidine hydrochloride (0.3 mg/kg), midazolam (4 mg/kg), and butorphanol tartrate (5 mg/kg). The mice were then sacrificed by cervical vertebra dislocation under anesthesia, and the intestine and clumps of Colon26/Luc cells in the peritoneal cavity were excised and then homogenized with lysis buffer (0.2 μM EDTA-2Na, 0.1 M Tris, and 0.8 μM Triton-X, pH 7.8). The homogenates were centrifuged at 15,000 rpm (Kubota 3500; Kubota, Osaka, Japan) for 5 min, and the supernatants were used for luciferase assay. Luciferase activity was measured by a luminometer (Lumat LB 9507; EG & G Berthold, Bad Wildbad, Germany) after addition of the luciferase assay buffer (PicaGene; Toyo Ink, Tokyo, Japan) and expressed as RLU per gram of tissue. One mouse that had been treated with the MDM2 siRNA complex was excluded due to the failure of intraperitoneal transplantation resulting in the proliferation of Colon26/Luc cells subcutaneously.

The survival rate of the mice treated with the complex was also monitored up to 60 days after inoculation with the Colon26/Luc cells (*n* = 5).

### 4.10. Statistical Analysis

Statistical analysis was performed by JMP 16 software (SAS Institute Inc., Cary, NC, USA). Dunnett’s pairwise multiple comparisons *t* test was performed for multiple comparisons among the groups. The log-rank test was used to compare the survival curves. Values of *p* < 0.05 were accepted as indicating statistical significance.

## Figures and Tables

**Table 1:** Particle sizes and ζ-potentials of siRNA complexes.

Complex	Size (nm)	ζ-Potential (mV)
MDM2-siRNA complex	177.1 ± 1.7	−34.9 ± 0.5
Scramble-siRNA complex	172.5 ± 4.0	−35.3 ± 1.0

Each value represents the mean ± S.D. (*n* = 3).

**Table 2:** Primer sequences used for real-time PCR.

Primer	Forward Primer Sequence	Reverse Primer Sequence
MDM2	5′-AGCACCTCACAGATTCCAGC-3′	5′-GCGCTCCAACGGACTTTAAC-3′
GAPDH	5′-TCTCCTGCGACTTCAACA-3′	5′-GCTGTAGCCGTATTCATTGT-3′

## Data Availability

The original contributions presented in this study are included in the article. Further inquiries can be directed to the corresponding author.
